# Single Photon Avalanche Diodes: Towards the Large Bidimensional Arrays

**DOI:** 10.3390/s8084636

**Published:** 2008-08-06

**Authors:** Simona Privitera, Salvatore Tudisco, Luca Lanzanò, Francesco Musumeci, Alessandro Pluchino, Agata Scordino, Angelo Campisi, Luigi Cosentino, Paolo Finocchiaro, Giovanni Condorelli, Massimo Mazzillo, Salvo Lombardo, Emilio Sciacca

**Affiliations:** 1 INFN-Laboratori Nazionali del Sud, via Santa Sofia 65, 95125 Catania (Italy); E-mails: sprivitera@lns.infn.it, tudisco@lns.infn.it, lanzano-l@lns.infn.it, fmusumci@lns.infn.it, campisi@lns.infn.it, cosentino@lns.infn.it, finocchiaro@lns.infn.it; 2 DMFCI-Dip. Met. Chim. Fis. Ing. & Dip. di Fisica ed Astronomia, Università di Catania viale A. Doria 6, 95125 Catania, (Italy); E-mails: pluchino@ct.infn.it, ascordin@dmfci.unict.it; 3 R&D, ST-Microelectronics, Stradale Primosole 50, 95100 Catania (Italy); E-mails: giovanni.condorelli@st.com, massimo.mazzillo@st.com; 4 IMM-CNR, Stradale Primosole 50, 95100 Catania (Italy); E-mails: salvo.lombardo@imm.cnr.it, emilio.sciacca@imm.cnr.it

**Keywords:** Single photon, avalanche photodiode, silicon photomultiplier, photon detection efficiency, timing, afterpulsing, cross-talk, 2D array

## Abstract

Single photon detection is one of the most challenging goals of photonics. In recent years, the study of ultra-fast and/or low-intensity phenomena has received renewed attention from the academic and industrial communities. Intense research activity has been focused on bio-imaging applications, bio-luminescence, bio-scattering methods, and, more in general, on several applications requiring high speed operation and high timing resolution. In this paper we present design and characterization of bi-dimensional arrays of a next generation of single photon avalanche diodes (SPADs). Single photon sensitivity, dark noise, afterpulsing and timing resolution of the single SPAD have been examined in several experimental conditions. Moreover, the effects arising from their integration and the readout mode have also been deeply investigated.

## 1. Introduction

In the last three decades several research teams investigated the possibility to build a silicon photosensor suitable for single photon counting applications ([1] and references therein). The original idea firstly proposed by R.J. Mc Intyre [2] was to implement a semiconductor photodiode with characteristics suitable for the triggered avalanche operation mode and therefore able to detect single photons [3-6] (whence the name Single Photon Avalanche Diode - SPAD). When a *p-n* junction is inversely biased 10 ÷ 20 % above the breakdown voltage value, a single charge carrier entering inside the high field region of the depleted volume can trigger the avalanche multiplication process (operating in Geiger mode). The fast leading edge (rise time less then 1 ns) of the corresponding current pulse can be used for detecting and timing the photo-generated carriers. In that condition, due to the “detectable” value of the flowing current, single optical photons can be detected.

Diode current is negligible until the first carrier enters or is generated in the junction depletion layer; a suitable circuit, usually called quenching circuit (passive or active), senses the rise of the diode current and quenches the multiplication process by lowering the bias voltage down below the breakdown [17].

To be used as SPAD, a diode must have a structure that fulfills some basic requirements: *(i)* the breakdown must be uniform over the whole active area in order to produce a standard macroscopic current pulse; *(ii)* the dark counting rate must be sufficiently low; *(iii)* the probability to generate afterpulses should be low. Operating in a dark environment, the carrier sources are essentially two: the diffusion current by quasi neutral regions, which is normally negligible [7], and the generation of electrons or holes from trap levels located in the depletion layer. In order to satisfy *(ii)* and *(iii)* prescriptions, both the effects of thermal carrier generation and trapping should be minimized.

A complete characterization of the SPAD device require an estimation of some important figures of merit: dark counting rate (thermal and afterpulsing components), photon detection efficiency, time resolution, maximum excess bias voltage, optimal working temperature, etc.

Today modern technology gives also the possibility to produce SPAD detectors with an integrate quenching mechanism based on a Metal-Resistor-Semiconductor structure. Precise resistive elements are embedded for each individual micro-cell of the array and provide effective feedback for stabilization and quenching of the avalanche process [8]. Such technology allows the production of large numbers of micro-cells on a common substrate (with or without a read-out circuitry) in order to achieve new imaging devices [27] or high-resolution and high-sensitivity Silicon Photomultipliers (SiPM) [9,26,28]. In this paper we present the results of a characterization work on prototypes of the next generation devices.

## 2. The fabrication process

**Figure 1.**
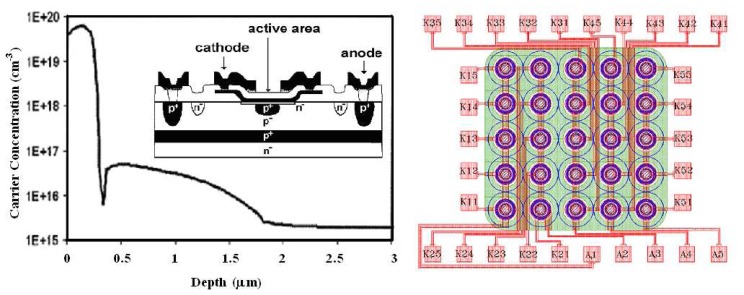
*(left)* Vertical cross-section of the SPAD device and the profile of carrier concentration inside the junction. *(right)* Layout of the 2-D array of 5×5 SPAD devices with the particular active area of 20 μm of diameter.

Figure 1 (left) shows the cross-section of the SPAD structure, as manufactured by ST-Microelectronics in Catania. The process starts with a *Si* <100> *n*^-^ substrate on which is grown a boron doped epitaxial layer with a *p*^+^ buried layer and with a *p*^-^ doped layer. The reason to form a buried *p-n* junction is twofold. First, the detector time response is improved because the effect of photo-generated carriers diffusing in the undepleted region is reduced [10]. Second, isolation from the substrate is introduced and makes it possible the integration of various SPADs and their circuitries. The *p*^+^ buried layer is necessary to reduce the series resistance of the device. The *p^-^* layer must be thin enough to limit the photo-carrier diffusion effect above mentioned. A good tradeoff has to be found for this thickness, because if it is made too thin the edge breakdown occurs at a voltage not much higher than the breakdown voltage of the active area. In order to reduce the contact resistance of the anode and provide a low resistance path to the avalanche current, the *p*^+^ sinkers are then created with a high-dose boron implantation step.

The next step, consisting on a local gettering process, is a key step in the process and was introduced in the last recipe. At this point of the process a heavy *POCl_3_* diffusion through an oxide mask is made on the topside of the wafer close to the device active area. Heavy phosphorus diffusions are well known to be responsible for transition metal gettering [11]. Unfortunately, the well-known phosphorous pre-deposition on the backside of the wafer is not able to getter the distant active area of the device because metal (*Pt*, *Au*, *Ti*) too slow during the final anneal. For this reason, if the gettering sites are created suitably close to the active region, a major improvement is observed.

The next step is the *p*^+^ enrichment diffusion obtained with a low energy boron implantation, producing a peak concentration of 5×10^16^ cm^-3^, followed by a high temperature anneal and drive in [12]. The first generation of devices was fabricated with a deposited polysilicon cathode doped by Arsenic implantation and diffusion. In order to damage as little as possible the active area of the device, the *As*^+^ ion implantation energy was carefully calculated; nevertheless, devices with very high dark-counting rate have been obtained. A remarkable improvement was obtained in the second generation by doping in situ the polysilicon. Further improvement was achieved in the third generation by accurately designing a Rapid Thermal Anneal to create a precisely controlled shallow Arsenic diffusion below the polysilicon in the *p*-epilayer. The final net doping profile has been measured by spreading profiling and it is shown in figure 1 (left). An important issue for the high SPAD quality is the uniformity of the electric field over the whole active area. If the electric field is not uniform, the photon detection efficiency (PDE) of the device becomes dependent on the absorption position on the active area. The lower the electric field the lower the PDE. Quality of the manufactured photodetectors has been checked by means of Emission Microscopy measurement [13].

Planar view of an array manufactured by integration of 25 pixels in square geometry 5X5 [13] is reported in figure 1 (right). Pixels of three different diameter dimensions, 20, 40 and 60 μm, have been chosen for the integration on the same structure. Separation distances between adjacent pixels, according to different diameters, ranges between 160 μm and 240 μm. Anode contacts are in common for each row, whereas each cathode is separately contacted and available to the external by different pads. One difference concerning the vertical structure of the array, respect to the single pixel construction, is the gettering region which is uniformly surrounding the active area of the pixel. Breakdown voltages distribution over an array has a mean value of 31*V* for the 20 μm device with a standard deviation of 0.08 *V*. In order to study the reduction of the optical cross-talk contribution, arrays that are optically and electrically isolated by deep thin trench technology have been already designed and fabricated. The trench process starts with a vertical etch 10 μm deep and 1μm large, a subsequent oxide deposition for complete electrical isolation. The process continues with tungsten filling, to avoid optical cross-talk and ends with planarisation. As it will be shown from measurements reported in the next sections, the lower average dark count rates indicate that trenches processing had an efficient gettering function.

## 3. General features

SPAD operating conditions require a bias supply voltage exceeding junction breakdown voltage of an amount called excess bias voltage (EBV) or over-voltage; such quantity has fundamental influence on the detector performance. A photon is detected if it is absorbed inside the sensitive volume and, due to the very high field, if the primary generated carriers trigger the avalanche multiplication process. Since a higher electric field enhances the probability to trigger the avalanche, photon detection efficiency increases with the EBV, PDE measured at room temperature, for 20% of EBV was about: 50% at 550 nm, 10% at 850 nm and 3% at 1000 nm [14].

SPAD operating conditions require also a suitable circuit, usually called quenching circuit (passive or active), which senses the rise of the diode current and quenches the multiplication process by lowering the bias voltage down below the breakdown. The features of the quenching circuit dramatically affect the operating conditions of the detector and, therefore, its actual performances. Since the ST-technology gives the possibility to realize SPADs with integrated resistors we will focus our attention on passive quenching technique.

The Passive-Quenching Circuit, PQC, was widely discussed in ref. [16, 17]: the avalanche current is quenched itself by increasing a voltage drop on a high impedance load. The SPAD is reverse biased trough a high ballast resistor *R_B_* of 100 KΩ or more, the junction capacitance value is typically a few hundred of fF, and stray capacitance (to ground of the diode terminal connected to *R_B_*, typically a few pF. The diode resistance *Rd* depends on the semiconductor device structure, and is of the order of some hundred of Ωs. Avalanche triggering corresponds to closing the switch in the diode equivalent circuit. The avalanche current discharges the capacitance so that diode voltage and diode current exponentially fall [15]. Avalanche quenching corresponds to the opening of switch in the diode equivalent circuit [17]. Small currents in ballast resistor *R_L_* slowly recharge the capacitances; the diode voltage exponentially recovers toward the bias voltage. A photon that arrives during the first part of the recovery is almost certainly lost, since the avalanche triggering probability is very low. The output pulse from a PQC can be obtained by inserting a low value resistor *R_L_* in series (50 Ω) on the ground lead of the circuit; in a such condition the pulse waveform is directly determined by the diode current.

A typical (anodic) signal from the passively quenched SPAD devices is characterized by a very fast climb up to a maximum positive value followed by a slow tail; the avalanche discharges the capacitance so that the diode voltage and diode current exponentially fall toward the asymptotic steady-state values of *V_f_* and *I_f_* [17]. The fast rising time of the SPAD pulse, about few hundreds of picoseconds, is due to the avalanche formation, which is connected to the intrinsic characteristics of the diode. On the other hand, the successive exponential fall time is drove by the quenching mechanism; quenching time constant *t_q_* was set by the total capacitance and by *R_d_* and *R_B_* in parallel (in practices simply by *R_d_*).

The maximum amplitude of the signal depends on both the series resistor *RL* and EBV; typical value was about 40 mV for 10% of EBV.

**Figure 2.**
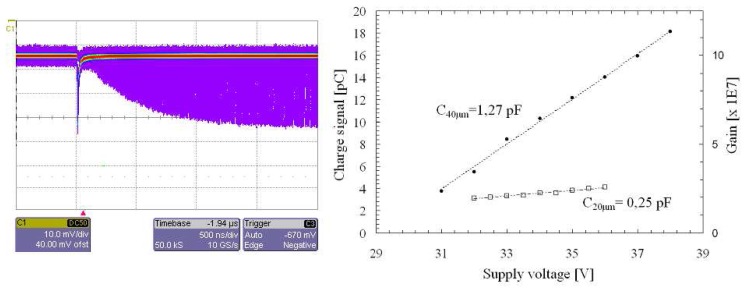
*(left)* Correlation between the maximum amplitude SPAD signals obtained in full bias conditions and the signals obtained during the successive recovery phase. The figure, obtained in persistence mode of the oscilloscope (after inversion of their polarity), shows a convolution of the pulses. Electrical pulses arise from a 40 μm passively quenched device, at 25 °C, with a 300 kΩ ballast resistor *R_B_* and biased at about 10% of EBV. *(right)* Capacitance values and the calculated gain for devices of 20 and 40 μm on active diameters.

After the quenching, the device exponentially recovers towards the original operating bias conditions. During this time a successive pulse shows a minor amplitude (see pulses convolution in figure 2 left). This recovery was set by the total capacitance and by ballast resistor *R_B_ (t_r_*, characteristic time constant) and remains approximately the same varying both temperature and excess bias voltage. Such feature may affect the performances of the device only in those applications which require very high counting rate.

Total capacitance and gain values were extrapolated by the linear fit of the charge accumulated and measured during the flow of the avalanche current as a function of the bias voltage, for both 20 μm and 40 μm devices. As shown in figure 2 (right), capacitances of 1.27 pF and 0.25 pF and their related gains were extracted.

**Figure 3.**
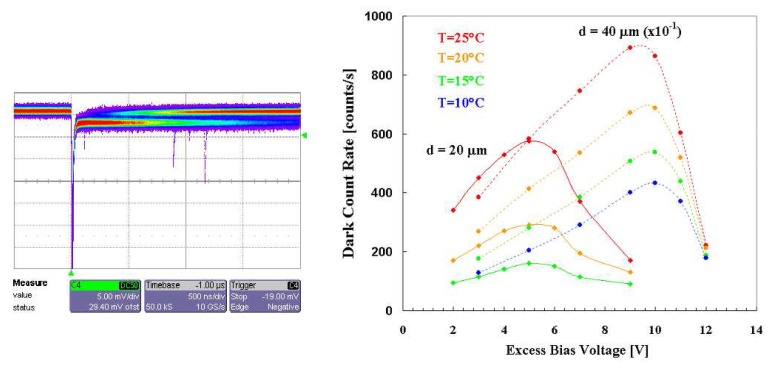
*(left)* SPAD signals on the digital oscilloscope (persistence mode), from a 20 μm passively quenched device, at 25 °C, biased at about 25% of EBV. At this EBV value there occurs a quiescent condition (see text). *(right)* Dark count rate as a function of the excess bias voltage, measured for passively quenched SPAD devices of active area with diameters d=20 μm (◆) or d=40 μm (●), at different temperatures: (dotted) T=25°, (dashed) T=20°, (solid) T=15°, (dash-dotted; T=10°. In order to plot both the set of data, counts arising from the bigger device was reduced a factor 10.

Another peculiar feature arising from the study of the pulse profile was the occurrence, at higher EBV, of a quiescent condition, characterized by a widely jittering duration. This is shown by the convolution of many signal tails reported in figure 3 (left). Such effect acted as a delay of the quenching mechanism and was a function of the EBV, being related [17] to the balance between *I_f_* and *I_q_* (a latching current). The avalanche is self-sustaining for *I_f_* above the latching current level and is self-quenching below it. Therefore, fixing the value of the ballast resistor, the transition was set by the EBV value, because *I_f_* =*EBV/R_B_*. As a general rule ballast resistor should be sufficiently high (50-500 KΩ); to perform our characterization we decided to work with 100 KΩ in order to reduce the total recovery time. For high values of EBV this effect results in an increase of the total dead time of the device, affecting also the measured trend of the dark counting rate versus the EBV (see figure 3 right). In particular when the EBV is too “high” (greater than 5 and 9.5 *V* for the 20 and 40 μm device respectively), the total quenching time can exceed the average time interval between two consecutive dark events, resulting in an apparent reduction of the measured dark count rate. On the other hand, under such limits, the dark count rate linearly increases with the EBV, because of two effects: *i)* the field-assisted enhancement of the emission rate from generation centers and *ii)* the increase of the avalanche triggering probability [18].

Thermal generation effects produce current pulses which represent the internal noise of the detector. As previously mentioned, the detector noise is due to both *i)* the Poissonian contribution of the dark counts arising by the thermal carriers generation, and *ii)* the occurrence of delayed pulses, due to the trapping and the delayed releasing of the avalanche carriers from deep levels inside the junction. Those released carriers have a certain probability to trigger other avalanches; the so-called afterpulsing may therefore affects the photon counting.

Temperature effects have been investigated by using a dedicated cooling system, which stabilizes the temperature of the package where the sample detector is mounted. A set of measurements of dark counting rate (pixel by pixel) operated with a scaler, as a function of both the EVB and the temperature, have been performed and reported for both the 20 and 40 μm devices in figure 3 (right). Typical measured values of dark counting rate at room temperature and at 10 ÷ 15% of EBV were: 400 cps and 2000 cps for the 20 μm and 40 μm pixel respectively. We observed the expected enhancement of the dark counts with both the temperature and the detector dimensions; the increasing with the temperature is connected to the intrinsic nature of the dark counts.

We remark that, in order to deeply evaluate the effect of temperature on dark counting rate, it is necessary to deeply investigate the afterpulsing contribution.

## 4. Afterpulsing

During the avalanche process some carriers may be captured by deep levels of the depletion region and subsequently released with a statistically fluctuating delay, whose mean value depends on the levels actually involved in the process [18]. Released carriers may re-trigger the avalanche and generate afterpulses correlated with a previous one. The number of trapped carriers during the avalanche increases with the total number of carriers crossing the junction and then with the avalanche current. Thus, these so-called afterpulses increase with both the delay of the avalanche quenching and the current intensity. Especially in the passive quenching strategy, the avalanche current is proportional to the EBV, which is chosen in order to perform the best operative conditions in terms of photon detection efficiency and/or timing performance [14, 15]. An alternative method to minimize the number of trapped charges per pulse requires a dedicated active circuitry, which acts on the quenching delay and reduces the current flowing across the junction.

A suitable technology must reduce both the generation and recombination centers to a very low concentration level and minimize the concentration of trapping levels. An appreciable improvement on the presented SPAD device has been observed with *(i)* the substitution of the in situ *n*-doped polysilicon layer to the implanted one and *(ii)* a local gettering process. Due to the uniform defect concentration over the device volume, a linear trend of the enhancement of dark counting rate versus active area has been measured in tests carried out on 5×5 arrays [19].

**Figure 4.**
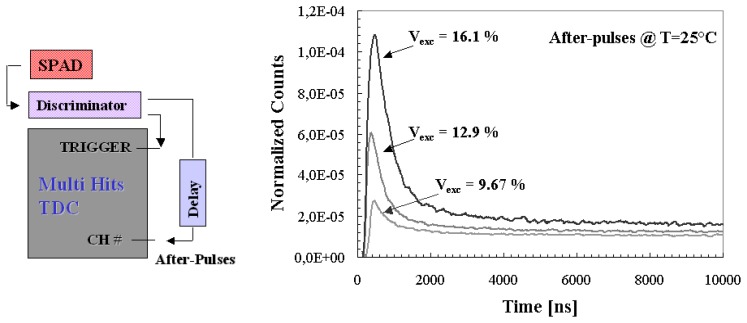
*(left)* Experimental set-up used for the afterpulsing measurements. *(right)* Timing distribution of the start-correlated events, for the three particular values of excess bias voltage and at room temperature. Counts were normalized to the total number of triggers.

An evaluation of the afterpulses contribution on passively quenched 20 μm devices, has been performed by using a new 32 channel multi-hit Time to Digital Converter (TDC), mounted on VME bus and part of a data acquisition system realized with standard nuclear electronics. Such TDC module is able to collect successive events within 50 μs, with a sensitivity of 100 ps. The detector signal was sent to a Constant Fraction Discriminator (CFD) and its output to the TDC module (see figure 4 left). The whole system was arranged in order to collect the afterpulses succeeding a reference trigger signal (start), given in our case by a dark event [18].

Measurements were performed in order to investigate temperature and EBV dependences on the after pulses. Some results are presented in figure 4 (right), where are reported timing distributions of after pulses succeeding a primary avalanche (trigger), normalized to the total number of starts, for the particular cases of three values of EBV at a fixed value of temperature.

Such distributions are characterized by two contributions: the correlated start events, representing the effective afterpulsing distribution, and the uncorrelated background representing the pure thermal dark counting rate of the detector. Moreover in the first hundreds of nanoseconds, there was observed also the contribution of the quenching and the successive recharging phase, where the pulses recover their original amplitude and, to be counted, must exceed the discriminator threshold.

**Figure 5.**
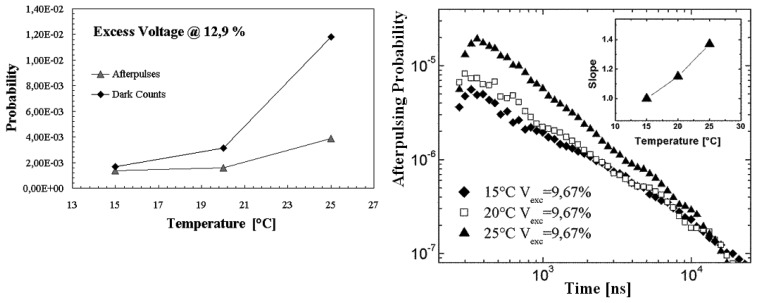
*(left)* Temperature trends of total afterpulsing probability and the dark counting probability in 10 μs window (biased was fixed at 12.9% of EBV and the discriminator threshold at 10 mV). *(right)* Timing distribution of the afterpulsing probability for different temperatures and fixed EBV (9.67%).

A comparison between dark counting probability and total afterpulsing probability, was performed through the subtraction of the uncorrelated background and a successive integration of the distributions in a 10 μs window; table 1 and figure 5 *(left)* show the results of such procedure. As expected, we observed a temperature and EBV dependence and, in particular, a less steeper decreasing of the afterpulsing contribution compared to the thermal dark count.

A further aspect arises from the logarithmic plot of afterpulsing distribution as a function of the temperature (figure 5 right). In the observed time window, the distributions were characterized by a power law trend with a slope parameter, which was evaluated by a fitting procedure, that is dependent on the temperature. Such observations are consistent with the common explanation of the afterpulsing phenomenon, in which, during the avalanche process, some carriers may be captured by deep levels of the depletion region and subsequently released. Nevertheless the observed power law trend, which typically characterizes the relaxation time of complex systems [20], suggests a possible description of the subsequent carrier release in terms of a decay of quasi-continuous distribution of energy levels [21, 22]. In such a description every level is characterized by a lifetime and, as expected, the contributions of levels with long lifetimes correspond to lower temperature values.

In the insert the slope parameter (evaluated by fitting procedure) is reported as a function of the temperature.

## 5. Timing

Due to the working principle, avalanche photo-diodes usually provide excellent timing performances (few hundreds of ps). Such excellent characteristics are often improved by using “ad hoc” special fast quenching electronics [17]. As far as the first generation devices, tested by using a simple active quenching circuit AQC [13], SPADs have also demonstrated such excellent performances. A future goal of the next generation of SPAD devices should be the large scale integration, therefore a compromise between timing performances and the needed simplicity of the used quenching circuitry on board is required. In this perspective some measurements devoted to the use of passive quenching technique, in different physical conditions, have been performed [15, 18].

**Figure 6.**
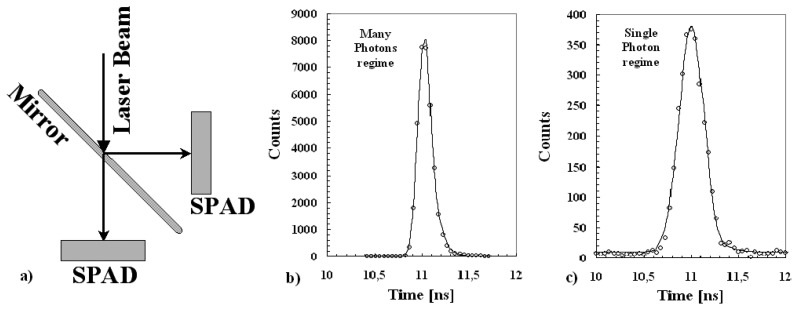
*a)* Experimental set-up; *b)* time jitter of SPAD with PQC in many photons regime, FWHM @ 0.161 ns; *c)* time jitter of SPAD with PQC in single photon regime, FWHM @ 0.31 ns

The employed set-up is reported in figure 6a. It was designed in order to guarantee the transition from many to single photon regime and in order to asses the achievement of single photon regime.

An optical pulse from a laser (declared time resolution, 35 ps FWHM, pulse energy 4 pJ) with two wavelengths 408 nm and 670 nm, used at 10 kHz of maximum repetition rate, was sent, via a semireflecting mirror, onto identical SPADs (40 μm active diameter and at 15% of EBV). The transition from the many to the single photon regimes was realized by using a gray filter (with a transmission coefficient of 0.01 %). A simple electronic chain, based on: a linear Fan-in Fan-out (to reverse the signal polarity) a CFD (Constant Fraction Discriminator), a delay module and a TDC (Time Digital Converter) has been used. The TDC start was taken from the laser trigger-out and the individual stops from the signals of each detector.

The transition between many to single photon regime was assessed by checking the rate of coincidence events between the two SPADs detectors. In fact, if is negligible the probability to have two photons, in the same event, in both SPADs, for symmetry reason, it will also be negligible the probability to have two photons on each detector. The use of 0.01 % filter guarantees such condition and the observed rate of coincidences was very low (<10^-3^).

The obtained results are synthesized in figure 6b-c and in table 2. An excellent time resolution of the SPAD was deduced also in this simple conditions. Timing spectra of the SPADs obtained in both single an many photons regime are well reproduced by a Gaussian plus an exponential fit. The exponential tail of Fig. 6b was dependent on the photons wavelength and in particular on their penetration depth (as discussed in previous works [1]). It is important to stress that the measured timing distribution in single photon regime includes also the laser timing structure.

### 5.1. Timing profile measurement with SPAD detector

In order to prove the real SPAD feasibility, a timing structure measurement of laser pulse was performed. Measure was performed by using: an UV pulsed laser (337 nm wavelength) with a declared timing resolution of 2 ns (FWHM) and a maximum repetition rate of 30 Hz, coupled with a dye laser system for the wavelength shift up to 395 nm.

**Figure 7.**
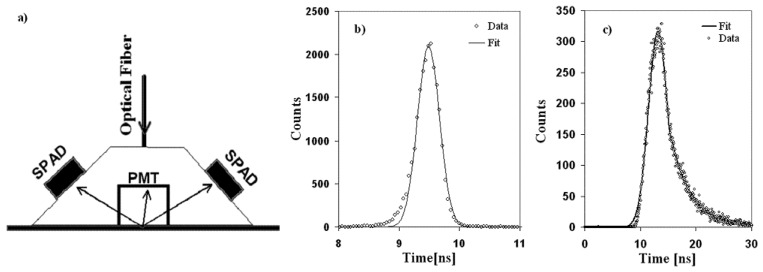
*a)* Experimental set-up; timing measurement of the pulses coming from a nitrogen-dye laser in: *b)* many photons regime (time jitter) *c)* single photon regime (dye laser timing profile).

To validate the effectiveness of our detection systems the measurements have been performed in both many and single photon regimes. The used set-up is reported in figure 7a: two identical SPADs of 40 μm in active diameter, were coupled to the two opposite faces of a trapezoidal shaped piece of Plexiglas. As reference standard readout detector, an HAMAMATSU R6427, 20 mm diameter PMT was coupled. We stress that the optical coupling of the photo-sensors was simply done by putting them in contact with the Plexiglas, as we were not aiming at the optimization of the yield. On the contrary, in order to operate also in the single photon regime, gray filters were used. The geometrical efficiency of each SPAD with respect to the PMT was of the order of 10^-5^. The electronic chain used was the same previously mentioned.

In order to assure the SPADs effective operating conditions, the single photon regime was checked by the evaluation that in no case, within our statistics, no events corresponding to the coincidence of the two SPADs with the PMT were present.

In figure 7b-c the timing spectra measured by one of the two SPADs in the different regimes, selected by using different filters, are reported (for more details see [24]). The spectrum obtained in the single photon regime (see Fig. 7 c) was perfectly reproduced by a gaussian plus exponential fit, reflecting the timing profile of the laser pulse, due to the dye excitation. In fact, in these conditions, the SPAD was triggered by photons generated at any instant within the light pulse whose time structure we reproduced. On the other hand, when detectors operate in many photons regime, the photon triggering the SPAD is typically the earliest and the obtained spectrum is a Gaussian whose width is determined by two main contributions: the detector resolution and the laser timing jitter, which is the dominant part.

## 5. Bi-dimensional arrays

Most of the important and current goals of photonics require the realization of large bi-dimensional arrays. With this aim a first array prototype, with 25 identical devices, in square geometry (5X5), was designed and manufactured [8]. The integrated devices were designed with a circular active areas and diameters of 20, 40, 50 and 60 μm and center to center distances between adjacent elements ranging from 160 to 240 μm. The wiring was realized as in the figure 1 (right), cathodes (tagged “*K#*”) were separately contacted and all the five anode contacts of each array were connected giving common rows, tagged “*A#*”, for the readout of the signal.

Unlike the single element device, in the array configuration the local gettering region uniformly surrounds the active area of each pixel through an external ring doped by heavy phosphorus diffusion, which also provides the decrease of the dark counting rate. In order to reach the uniformity of the breakdown within the entire active area, a virtual guard ring using a large window of *n*^+^ polysilicon was created; moreover, a good uniformity (less then 1%) on the whole device of the array was found. A sampling on dark counting rates on 30 equal arrays of 5×5 equal elements with an active area of 20 mm in diameter has shown a very narrow statistical distribution with an average value of about 400 cps and a dispersion of 50 cps [19].

Other limitations to the photon counting arise from the integration of adjacent devices: by the ignition of the avalanche process in a SPAD, spurious uncorrelated avalanches may be triggered in the neighboring devices, due to the optical and/or electrical induction. This so called cross-talk effect, increases the detector noise.

The avalanche multiplication process in a *p-n* junction reverse biased over the breakdown value, can lead to the production of secondary photons ([23] and reference there in) by radiative emission from the hot-carriers. This possibility can originate further avalanches in the near detectors. The emission probability was estimated in 10^-5^ photons per carrier crossing the junction, the attenuation length, in silicon, for near UV and visible photons was 80 μm. Such contribution represents the fast component of the cross-talk and may be minimized by both: a suitable optical isolation among the diodes (if the pixels are very closed); and reduction of the number of hot-carriers.

The electrical induction can occur when carriers, generated during the avalanche process, can overcame the junction, reaching and triggering the neighboring devices; this represents the slow component of the cross-talk.

A further contribution arise from the pixels wiring, it may become important when the density of implemented elements is high and the distances between the wiring become smaller.

In order to avoid the optical cross-talk, during the fabrication of arrays of SPAD a delicate process connects “trenches” with metal coated sidewalls (into the bulk of semiconductor) between pixels, reducing the minimum distance between elements and increasing the dynamic range of the device. This is a delicate process because metal is deposited close to the pixel after a difficult previous removing from the active region. Currently, arrays of SPAD optically and electrically isolated by deep thin trench technology have been designed and fabricated (see section 2).

Cross-talk analysis can be realized with different techniques [29], in the present work it was performed in some crucial steps; primarily the possible electric and/or electromagnetic contribution between two pixels was investigated by the signals analysis and their correlation on digital oscilloscope.

**Figure 8.**
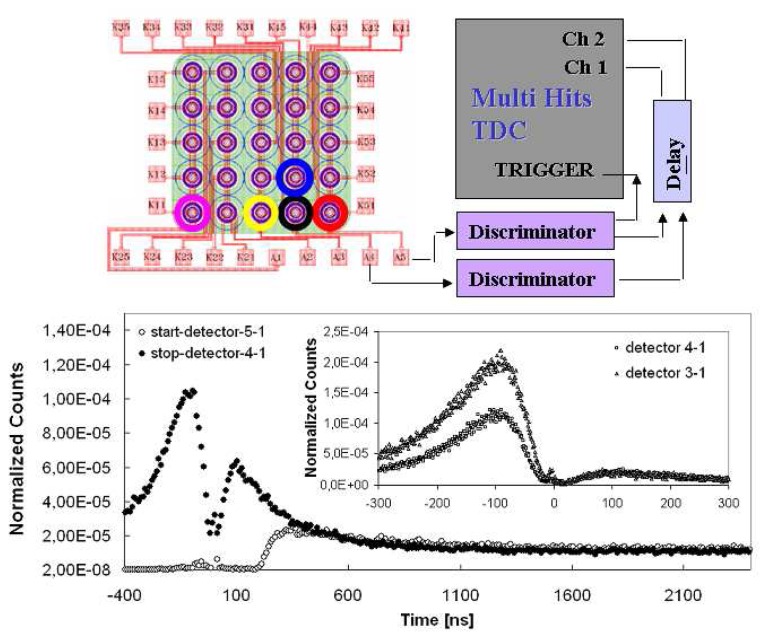
*(top)* Experimental set-up used for correlation measurements; trigger was realized with 5-1 pixel (red), correlation was studied in the 4-1 (black), 4-2 (bleu), 3-1(yellow), 1-1(pink) pixels. *(bottom)* Normalized Counts distribution for the pixels 5-1 (afterpulses) and 4-1 (inter-pixel correlations) obtained normalizing the Ch1 and Ch2 spectra to the total number of triggers (avalanches on the start detector). In the inset there are reported Ch2 spectra alternatively when the correlated detector was 4-1 or 3-1.

Following the layout reported in figure 8 *(top)*, one pixel of first column was chosen as a reference (its signal was used to trigger the oscilloscope, the measurements were performed with dark events) and the induced signals on both neighboring and far pixels were studied.

As expected from the high pitch values, samples with and without trench manifest the same behavior and in particular: *(i)* a small correlated pulse of opposite polarity compared to the reference signal with a similar timing structure was found; *(ii)* such correlated signal results quite independent from the inter-pixel distance. From this data the substrate, and its resistive value, seems to have e dominant contribution, it acts as a sort of propagator, inducing field fluctuations on the other pixels of array.

After these simple deductions a deeper investigation of the cross-talk was performed by means of the timing correlation measurements on a couple of array elements. Experimental set-up was arranged by using a 32 channel multi-hit Time to Digital Converter (m-TDC) module which were a part of a data acquisition system based on standard nuclear electronics. TDC inputs, was taken from the array elements (see figure 8 top). The whole system is able to correlate signals in 50 μs window starting from a master trigger which, in our case, was generated by a dark event in the pixel chosen as a reference. The two detectors signals were also delayed and sent to the TDC channels in order to: (Ch1) follow the afterpulsing dynamics of the reference detector and (Ch2) study the correlations. To check the validity of the method and the operating conditions a preliminary test with two uncorrelated pixels of two different arrays was performed. As expected, the correlation spectrum (Ch2) was completely uncorrelated. The element 5-1 of the 5×5 array was chosen as reference detector (Ch1) and alternatively, in order to investigate the inter-pixel distance dependence, the pixels 4-1, 4-2, 3-1 and 1-1 as the second detector (Ch2). In a such way the events on Ch1 were the afterpulses of the reference detector whereas events on Ch2 were the cross correlations.

In figure 8 *(bottom)* are reported Ch1 and Ch2 spectra (events normalized to the total number of acquisition starts) related to the pairs 5-1→4-1 and 5-1→3-1 (see also the insert of figure 8 bottom); the Ch1 spectrum show both the two contributions of the uncorrelated dark events and the after pulses modulated in the first hundreds of nanoseconds from the quenching mechanism and the successive recharging phase, as discussed in section 4.

A similar structure, over the uncorrelated dark background, was observed on both the two temporal regions (negative and positive) of Ch2 spectrum; the zero correspond to the time of arrival of a start signal. The correlated events before such time were connected to the primary avalanches generated inside the pixel 4-1 which induce same correlation on the start (pixel 5-1). On the contrary, time positive events correspond to the true correlation of the detector 4-1 in respect to the 5-1 (start detector) where the primary avalanche was generated. The strength of such contributions seems to be quite similar to the observed afterpulsing correlation (Ch1 spectrum) without effects due to the quenching and successive recharging. Moreover, the cross correlated events reported in the inset of figure 8 bottom show a prompt contribution confined in the first nanoseconds with a total probability (after uncorrelated background subtraction) of the order of 5·10^-5^ which increases with the excess bias voltage [24] and connected to the first observations. The breakdown determines an alteration of the field conditions on the other detectors of the structure, inducing with a certain probability a further avalanche.

**Figure 9.**
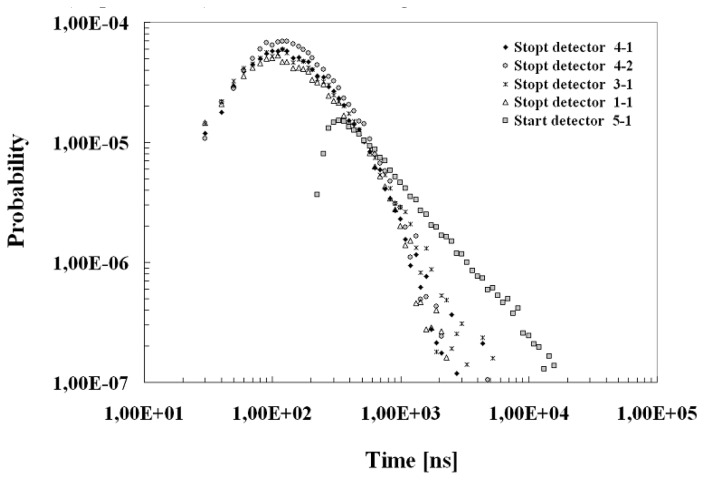
Afterpulsing probability distribution (start detector) and cross-talk probability distribution (stop detectors) obtained subtracting the uncorrelated dark events.

Concerning the slow part, measurement was repeated for several detector pairs and the obtained data were analyzed and treated as discussed in section 4. As illustrated in figure 9, we discovered a substantial difference in the timing behavior of the afterpulses distribution with respect to the correlations. A faster kinetics seems to characterize the cross-talk distribution, without any significant difference with respect to the inter-pixel distance. Such phenomenon could be interpreted as due to a charge trapping phenomenon and successive release from the not active zones surrounding the pixels, probably the substrate, or due to the photo-production of pairs outside the active zone or due to luminescence effects.

## 6. SiPM concept

A photomultiplier based on the silicon technology represents the new frontier of photodetection. SPAD's integrated on the same substrate, with a common read-out, could satisfy such expectations. The proposed configuration is able to detect and count the photons arrival giving an output pulse directly proportional to the source intensity (like a photomultiplier), that was excluded for the single device operating in Geiger-mode. Moreover, by using Metal-Resistor-Semiconductor structures is possible to realize devices together with their integrated quenching circuitry; resistive elements are chosen and embedded for each individual micro-cell, providing the effective feedback for stabilization and quenching of the avalanche process [8, 9, 25, 26].

Such device can be obtained from the common readout of bi-dimensional SPAD arrays. The so-called SiPM should provide: high sensitivity to single photon counting, high quantum detection efficiency over a wide part of the spectrum (up to the near IR region), insensitivity to magnetic field and very low bias voltage.

**Figure 10.**
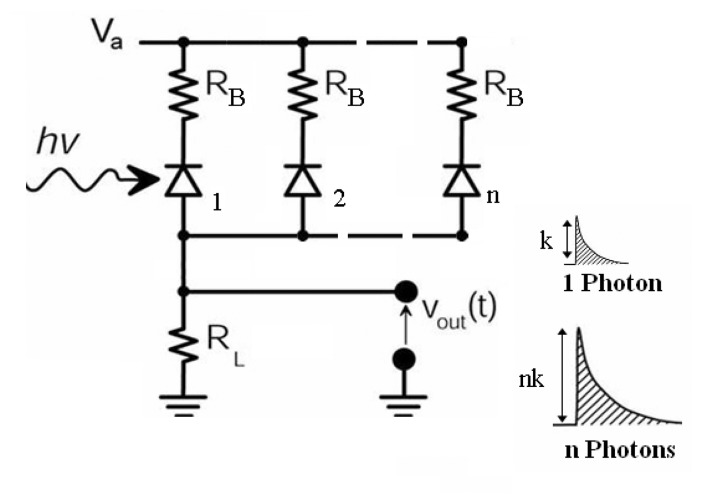
Simplified diagram of an array of SPADs in SiPM configuration.

In this section, the 5×5 SPAD arrays were arranged in SiPM configuration (fig. 10) and their performance will be presented. SiPM results from the parallel readout of every SPAD element, each one of which is passively quenched by means of a ballast resistor of 100 kΩ. The output signal is detected on the common load resistor *R_L_* which connects all the anodes to the ground. In this architecture, the signal is the sum of all the individual cells fired by the photon-initiated avalanche phenomenon. Each single SPAD element operates as a binary device, while their combination makes the device an analogue detector. The output current signal observed at the oscilloscope manifests a multiple structure with several amplitudes: for example, when two photons are simultaneously detected by two (different) pixels a signal with double amplitude was expected. Similarly, when *n* photons are simultaneously detected, it exhibits a multiple structure with several amplitudes.

**Figure 11.**
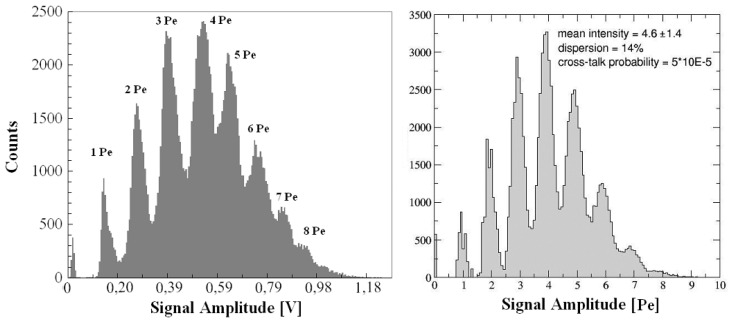
*(left)* Amplitude distribution of signals from the 5×5 array of SPADs once illuminated by a laser pulse of λ=670 nm. *(right)* Montecarlo simulation of signals amplitude distribution of 5×5 SPADs array in SiPM configuration once illuminated; See the text for the input parameters.

To test the whole device as a function of the number of impinging photons, the array was illuminated by a picosecond laser (λ=670 nm), adjustable in intensity and in repetition rate. The array was biased at 10% of the EBV, cooled at the steady temperature of 20 °C, the output signal was processed by using an amplitude to digital converter (ADC) and the results have been reported in figure 11. The measured amplitude distribution shows several peaks corresponding to the detection of a single (double, triple, etc.) photoelectron(s). Such results demonstrate, also in this hybrid configuration, the excellent performance of the device in terms of single photoelectron resolution.

These results were interpreted also by using the output of a Montecarlo simulation which reconstructs the amplitude signal distribution of array taking into account the single detectors response and the cross-talk effects. In particular such code simulates the amplitude distribution of signals from an array illuminated by a laser pulse, starting from the single pixel response in terms of profile, total duration, rise and fall time. The code takes into account also the non-uniformity of the pixels response. The input parameter were:

*(i)* the laser intensity and its dispersion, representing the number of fired pixels normally distributed *(ii)* the percentage of non-uniformity among all the pixels, essentially due to the hybrid configuration, external circuitry, solders, etc *(iii)* the cross-talk probability, assumed with an infinite interaction range *(iiii)* the dark counting rate.

**Figure 12.**
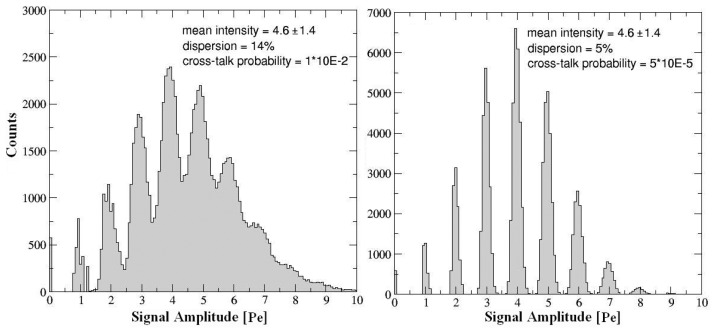
Montecarlo simulation of signals amplitude distribution of 5×5 SPADs array in SiPM configuration once illuminated; *(left)* cross-talk effects *(right)* dispersion effects.

The values of parameters that better reproduce the experimental distribution are reported in the figure 11 and 12; the extracted 14% of non-uniformity parameter was in reasonable agreement with the experimental observation. It appears that an increasing non-uniformity generates an asymmetric distortion of the amplitude distribution and the cross-talk acts on the Gaussian-like background shifting the distribution towards high photoelectron peaks. A technological migration toward a SiPM configuration with integrated quenching resistors will reduce the dispersion effects but not the cross-talk and the afterpulsing. A deeper understanding of these phenomena will be necessary in order to achieve high performance devices.

## 7. Conclusions

The really promising results obtained with single photon avalanche diodes (SPAD) in terms of PDE, dark counting rate, timing, after pulsing probability, demonstrate how SPAD is an ideal candidate among the existing single photon sensors.

Moreover the integration possibility, investigated with arrays of 5x5 elements, the good performance obtained in terms of dark counting rate uniformity and cross-talk contribution seems very promising, especially for the future imaging and SiPM realization.

## Figures and Tables

**Table 1.** Comparison between the total afterpulsing probability and dark counting probability in a 10 μs window.

*T[*°*C]*	*Excess Voltage [%]*	*Afterpulsing probability*	*Dark Events on 10 μs*
15	9,67	7,69E-04	1,40E-03
20	9,67	8,53E-04	2,75E-03
25	9,67	1,73E-03	9,96E-03
15	16,1	3,49E-03	1,80E-03
20	16,1	4,27E-03	3,75E-03
25	16,1	2,08E-02	7,19E-03

**Table 2.** SPAD time resolutions for the two regime and for the two illuminating conditions.

*wavelength*	*PQC*	*τ*	*PQC*	*τ*
*408 nm*	*0.16 ns*	*0.07 ns*	*0.31 ns*	*0.10 ns*
*670 nm*	*0.14 ns*	*0.24 ns*	*0.38 ns*	*0.68 ns*
	*Many photons regime*		*Single photon regime*	
